# Designing with Li_2_S in Lithium–Sulfur Batteries: From Fundamental Chemistry to Practical Architectures

**DOI:** 10.1002/smll.202513644

**Published:** 2026-01-25

**Authors:** Hyeona Park, Arcangelo Celeste, Shulin Wang, Chaiwon Lee, Yul Yang, Kaizhao Wang, Aleksandar Matic, Sergio Brutti, Shivam Kansara, Shizhao Xiong, Marco Agostini, Jang‐Yeon Hwang

**Affiliations:** ^1^ Department of Energy Engineering Hanyang University Seoul Republic of Korea; ^2^ Department of Chemistry Sapienza University Rome Italy; ^3^ Faculty of Material Science and Engineering Kunming University of Science and Technology Kunming China; ^4^ Department of Physics Chalmers University of Technology Gothenburg Sweden; ^5^ Department of Chemistry and Drug Technologies Sapienza University Rome Italy

**Keywords:** all solid‐state Li/S batteries, charge‐transfer kinetics, electrocatalytic interfaces, electrolyte/solvation engineering, lithium sulfide (Li_2_S) cathodes

## Abstract

Lithium‐sulfur (Li‐S) batteries deliver gravimetric energy densities considerably higher than those of conventional lithium‐ion systems while relying on low‐cost, earth‐abundant materials. Despite decades of progress, their commercialization remains hindered by intrinsic challenges such as the insulating nature of sulfur and lithium sulfide (Li_2_S), formation and dissolution of soluble polysulfides, and instability of lithium‐metal anodes. Among these, the use of Li_2_S as a pre‐lithiated cathode has redefined the landscape of Li─S chemistry by offering a pathway toward lithium‐free and anode‐free architectures that are compatible with the existing manufacturing infrastructure. This perspective revisits the Li_2_S electrochemistry from a conceptual and design standpoint. The perspective emphasizes multiscale strategies for atomic‐level catalytic engineering, mesoscale electrode architectures, and electrolyte–interface control, which collectively determine Li_2_S activation and reversibility. The perspective also examines emerging approaches that integrate Li_2_S cathodes with graphite, silicon, and solid‐state configurations to enable safe, high‐energy, and manufacturable Li─S technologies. Finally, this perspective discusses the evolving roles of redox mediators, machine learning‐based discovery, and sustainable synthesis in bridging the gap between laboratory breakthroughs and industrial viability. Collectively, these insights frame Li_2_S not only as an alternative, cathode, but also as a platform for reimagining Li─S electrochemistry in the post‐lithium‐metal era.

## Graphical abstract

This perspective highlights the design evolution of Li_2_S‐based lithium‐batteries, illustrating sulfur redox chemistry and Li_2_S activation. Emphasis is placed on catalytic interfaces, hierarchical carbon frameworks, and electrolyte‐solvation co‐design, enabling lithium‐free, anode‐free, and solid‐state Li‐S architectures for high‐energy, manufacturable systems.

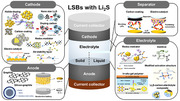

## 1 Introduction

### 1.1 From Molten Sodium‒Sulfur (Na─S) Cells to Lithium‒Sulfur (Li─S) Chemistry

Sulfur has always been centrally positioned in the development of electrochemical energy‐storage systems [1, 2, 3] Sulfur is widely available and is often recovered as an industrial byproduct of fossil fuel refining, making it economically attractive and environmentally advantageous [4, 5, 6]. The modern history of sulfur batteries began in the 1960s with molten NaS cells employing a ceramic β‐alumina solid electrolyte and operating at approximately 300–350°C [7, 8, 9]. These high‐temperature systems were the first large‐scale use of sulfur as an active cathode material, demonstrating the viability of sulfur for stationary storage. Concurrently, the redox versatility of sulfur inspired early Li─S concepts thanks to the exceptional theoretical capacity (1672 mAh g^−1^) [1, 2, 3]. Herbert and Ulam patented a Li─S cell in 1962 [10], and although these designs remained largely exploratory, they established the electrochemical couple:

(1) $$S_{8}+16\;\mathrm{Li}\;↔8\;Li_{2}S$$

Research conducted during the 1980s and 1990s clarified the intrinsic difficulties of Li‐S cells, that is, the insulating nature of both sulfur and its reduction product lithium sulfide (Li_2_S), the dissolution and migration of lithium polysulfides, and the approximately 80% volume expansion between sulfur (S_8_) and Li_2_S [11, 12, 13, 14]. The early 2000s brought major progress through carbon–sulfur composites, nanostructured hosts, and electrolyte additives such as LiNO_3_, which stabilized the Li/electrolyte interface [15, 16]. These developments transformed Li─S chemistry from a conceptual curiosity into a vibrant research domain promising theoretical energy densities > 2500 Wh kg^−1^, which is five‐ to six‐fold that of current lithium‐ion cells [17, 18].

### 1.2 Emergence of Li_2_S as a Cathode Concept

Strategies for enhancing the reactivity of Li_2_S have advanced rapidly since the early 2010s as the advantage of containing lithium to avoid pre‐cycling of sulfur electrode or counter‐anodic part. Nanoscale Li_2_S particles dispersed in conductive carbon matrices, Li_2_S–graphene composites, and heteroatom‐doped carbon hosts have dramatically improved electronic contact and polysulfide confinement [19, 20, 21, 22]. Parallel efforts in solid‐state configurations and semisolid slurries have leveraged the intrinsic lithiation of Li_2_S to avoid volatile liquid electrolytes [23, 24, 25]. Recently, operando characterization, density functional theory (DFT), and data‐driven screening have been used to elucidate the atomistic pathways of Li_2_S activation, linking interfacial catalysis to performance metrics. Transition‐metal phosphides, carbides, and sulfides embedded within carbon scaffolds have emerged as effective catalysts for lowering the decomposition barrier of Li_2_S and stabilizing intermediate species [26, 27, 28, 29, 30, 31, 32, 33, 34]. These developments collectively mark a transition in Li─S research, from mitigating polysulfide shuttling in elemental‐sulfur cathodes to engineering Li_2_S as an active, pre‐lithiated platform for safe, high‐energy batteries. The convergence of nanoscale design, catalysis, and interfacial chemistry defines the direction of the field. A close comparison between S and Li_2_S reveals important differences. Specifically, conventional S cathodes deliver a theoretical specific capacity of 1675 mAh g^−1^ (based on sulfur) while Li_2_S has 1166 mAh g^−1^. Despite the energy density, both gravimetric and volumetric, has been reported the same for the two materials, using Li_2_S as the starting cathode in lithium‐free anode systems improves gravimetric outcomes at the cell level compared with conventional Li─S cells that need excess lithium metal anode. Indeed, direct standalone theoretical gravimetric energy density of Li_2_S cathodes is not typically reported as a separate “higher” number than sulfur because sulfur's reaction to Li_2_S defines the Li─S system energy. Sulfur density ∼2.03 g cm^−3^ vs Li_2_S density ∼1.66 g cm^−3^ means conventional Li─S cathodes typically expand upon full lithiation (∼80% volume change) as S → Li_2_S, complicating electrode integrity and reducing effective density. Li_2_S cathodes start in the lithiated form, so volume change during cycling is less than for S cathodes, which can help maintain higher effective volumetric densities in engineered electrodes. From a cost perspective, sulfur is extremely low cost, whereas Li_2_S is substantially more expensive due to lithium content and synthesis routes. However, Li_2_S enables the use of lithium‐free anodes, which can offset system‐level costs and improve safety. In terms of manufacturing complexity, conventional S cathode benefits from being chemically stable to air and moisture and thus it can be handled under ambient conditions; however, it requires the use of Li‐metal, shifting handling complexity to the anode sidel.Li_2_S cathodes is highly sensitive to moisture, reacting to form LiOH and also releasing H_2_S gas, requiring more controlled electrode engineering, while it simplifies large‐scale cell assembly and improves manufacturing safety, due to the removing of metallic lithium and use of alternatives anodes (Si, Sn, etc.).

Based on these quantitative comparisons, we will clearly define the applicable scenarios: conventional sulfur cathodes are more suitable for cost‐sensitive applications where gravimetric energy density is prioritized, whereas Li_2_S cathodes are advantageous for high–volumetric‐energy‐density, safety‐critical, and lithium‐metal‐free battery systems, particularly in compact or automotive‐scale applications.

### 1.3 Evolution of Electrodes Design Strategies

**FIGURE 1:**
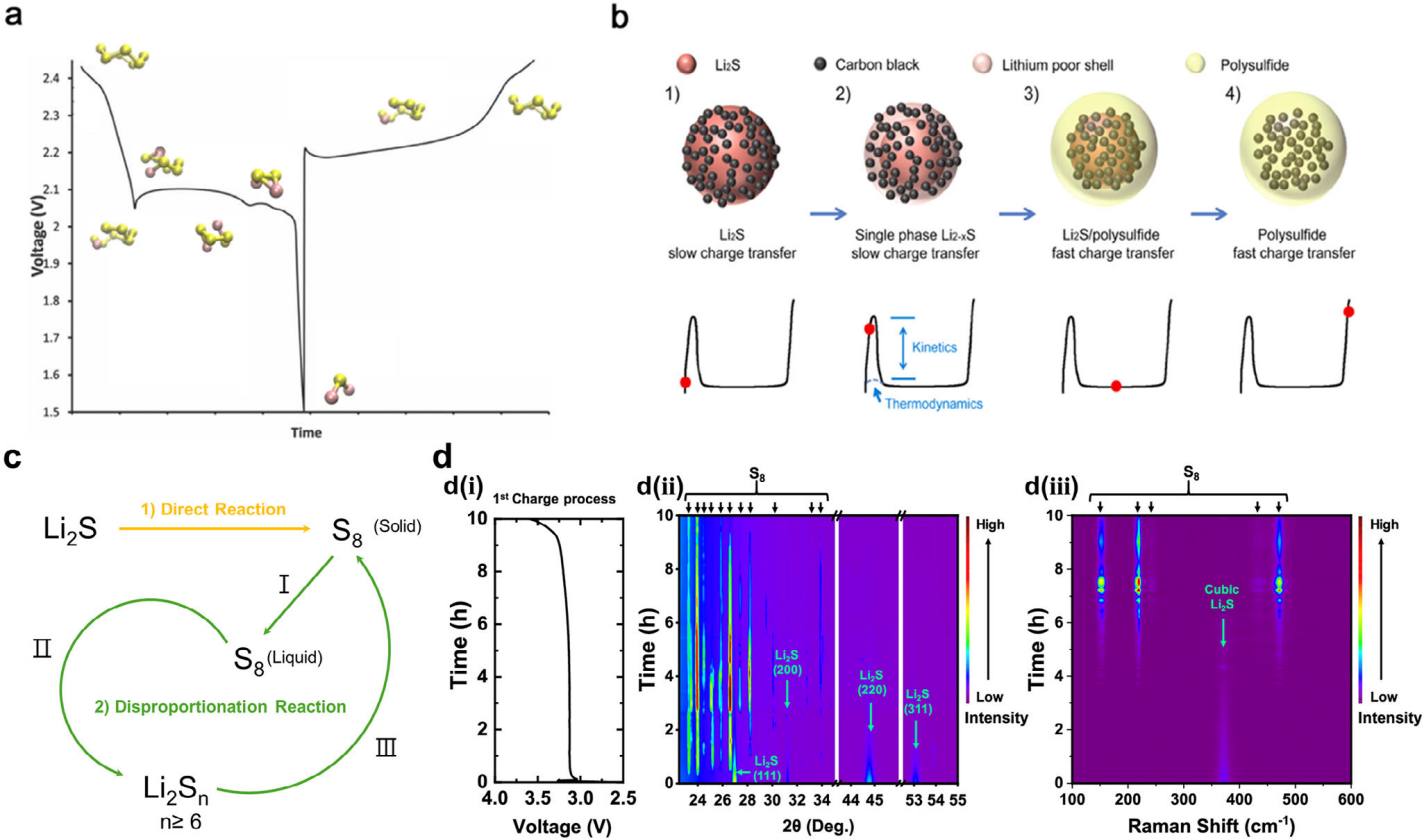
Comparison of the electrochemical processes of elemental sulfur and Li_2_S in the Li‒S system. a) Conventional discharge–charge mechanism of sulfur, involving the stepwise reduction of S_8_ through a sequence of soluble polysulfides (Li_2_S_8_ → Li_2_S_6_ → Li_2_S_4_) to the final insoluble product Li_2_S during discharging, followed by reverse oxidation during charging; Reproduced with permission [36]. Copyright 2008, Royal Society of Chemistry. b) First‐charge process of the Li_2_S cathode, showing the characteristic activation stage with a pronounced voltage barrier (∼1 V) associated with sluggish charge transfer and the nucleation of soluble polysulfide intermediates. Once activated, Li_2_S follows the same reversible redox pathway as sulfur, cycling through identical polysulfide phases; Reproduced with permission [35]. Copyright 2012, American Chemical Society. c) Schematic of reaction pathway of Li_2_S oxidation. d)  In situ XRD and Raman spectroscopy of pelletized Li_2_S/graphene composite cathode demonstrating direct conversion of Li_2_S to S_8_ in the first charge (d(i) First charge voltage profile d(ii) In situ XRD and d(iii) Raman spectroscopy); Reproduced with permission [37]. Copyright 2019, American Chemical Society.

Figure 1 summarizes the electrochemical processes of elemental sulfur and Li_2_S in the Li─S system. The electrochemical behaviors of sulfur and Li_2_S are fundamentally interconnected, as shown in Figure 1a. In conventional Li─S cells, S_8_ undergoes a multisteps reduction during discharging wherein S_8_ is converted into soluble long‐chain polysulfides (Li_2_S_8_ → Li_2_S_6_ → Li_2_S_4_) and finally into insoluble Li_2_S. Reverse oxidation occurs during charging. By contrast, the electrochemical activation of Li_2_S is dictated by two coupled kinetic barriers, as schematically shown in Figure 1b: (i) the energetic cost of extracting Li^+^ from an electronically insulating, highly ionic Li_2_S lattice; and ii) sluggish charge transfer across the Li_2_S/electrolyte interface.

Collectively, these produce the characteristic first‐charge overpotential for bulk Li_2_S, because Li^+^ extraction must coincide with the nucleation and solvation of higher‐order polysulfides whose formation/dissolution is strongly governed by the local solvation structure and interfacial chemistry. Once oxidation initiates, Li_2_S undergoes a solid→liquid transition to soluble Li_2_S_x_ (x > 2), which then participates as elemental sulfur (Figure 1b) [35]. The initial Li_2_S oxidation is kinetically sluggish, producing a large charge‐transfer resistance due to low Li^+^ diffusivity and formation of a lithium‐deficient surface layer (Figure 1, steps 1–2). Upon conversion to soluble polysulfides, charge transfer is markedly accelerated through liquid‐phase redox reactions (Figure 1, step 3). At full charge, only polysulfides remain(Figure 1, step 4). Alternatively, once the barrier is overcome, Li_2_S oxidation follows the same sequence of polysulfide intermediates that govern sulfur reduction, leading to a fully reversible reaction pathway consistent with the mechanism described by Wild et al. [36]. Therefore, S_8_ and Li_2_S represent opposite ends of the same redox couple, distinguished primarily by the kinetic constraints of Li_2_S activation. Otherwise, the initial delithiation reaction has been reported to proceed via direct conversion reaction (Figure 1c). The required overpotential is mainly due to differences on crystallization enthalpy. The pelletized Li_2_S/graphene composite cathode exists as highly deformed Li_2_S particles in a metastable state, enabling direct conversion (to S_8_). During the initial de‐lithiation process of Li_2_S, XRD and Raman spectroscopy analysis confirmed that no intermediate phase such as lithium polysulfides were observed (Figure 1d(i)) [37]. In situ synchrotron XRD collected during 1/10 C charging confirms the direct conversion of Li_2_S in the first charge (Figure 1d(ii)). Consistently, in situ Raman spectra show no detectable signals from soluble polysulfides (S_n_^2^^−^) during the initial charging stage (Figure 4(iii)). At a more fundamental level, this macroscopic activation barrier reflects coupled atomic‐scale energetic and electronic constraints intrinsic to Li_2_S. The pronounced first‐charge overpotential of bulk Li_2_S arises from coupled atomic‐scale energetic and electronic constraints. At the atomic level, Li extraction requires breaking strongly ionic Li─S bonds and enabling Li^+^ transport through the Li_2_S lattice or across the Li_2_S–host interface; nudged‐elastic‐band (NEB)‐DFT studies report substantial activation barriers for Li hopping and for the initial Li_2_S decomposition on inert carbon surfaces, which kinetically suppress oxidation until large overpotentials are applied [38, 39]. Electronically, Li_2_S is an extremely poor conductor and charge removal often proceeds through localized carrier formation (i.e. small polarons) and thermally activated hopping rather than band‐like transport; recent first‐principles work shows that the electronic localization and associated reorganization energies further increase the effective oxidation barrier [40]. At realistic electrode/electrolyte interfaces these atomic/electronic factors are modified by i) solvent/polysulfide solvation which stabilizes early oxidation products and can lower nucleation barriers, ii) surface adsorption and charge transfer to catalytic hosts that reduce the decomposition barrier of Li_2_S (DFT shows marked reductions on metal‐sulfide, phosphide and single‐atom M─N_x_ motifs), and iii) redox mediators that bypass direct Li_2_S electron removal by chemical oxidation pathways. Together, these mechanistic contributions rationalize why catalytic hosts, tailored solvation (LHCEs/weakly solvating cosolvents), or redox mediators reproducibly lower the first‐charge overpotential in experiments [41, 42].

### 1.4 From Conductivity Enhancement to Catalytic Design

Initial strategies in the late 2000s and the early 2010s aimed to overcome the poor conductivity and high activation barrier of Li_2_S. Nanoscale Li_2_S particles, Li_2_S‐carbon composites, and encapsulation methods were developed to enhance electronic pathways and improve electrolyte accessibility [43, 44]. Ball‐milling, solution‐phase synthesis, and electrospinning produced Li_2_S─C composites with homogeneous dispersion and improved interfacial reactivity [45].

Integration with graphene, mesoporous carbons, and conductive polymers further optimized electron/ion transport, enabling higher utilization and lower polarization [19, 20, 21, 22]. Simultaneously, Li_2_S has been incorporated into semi‐solid and all‐solid configurations to remove volatile electrolytes and enable lithium‐free architectures, [23, 24, 25]. Advanced operando characterization and computational modelling has clarified that the Li_2_S activation process is governed by coupled ionic extraction and electronic delocalization at the interface. This understanding catalyzed a shift from purely conductive frameworks toward functional catalytic composites capable of accelerating Li_2_S oxidation and stabilizing polysulfide intermediates.

## 2 Hierarchical Carbon Framework

**FIGURE 2:**
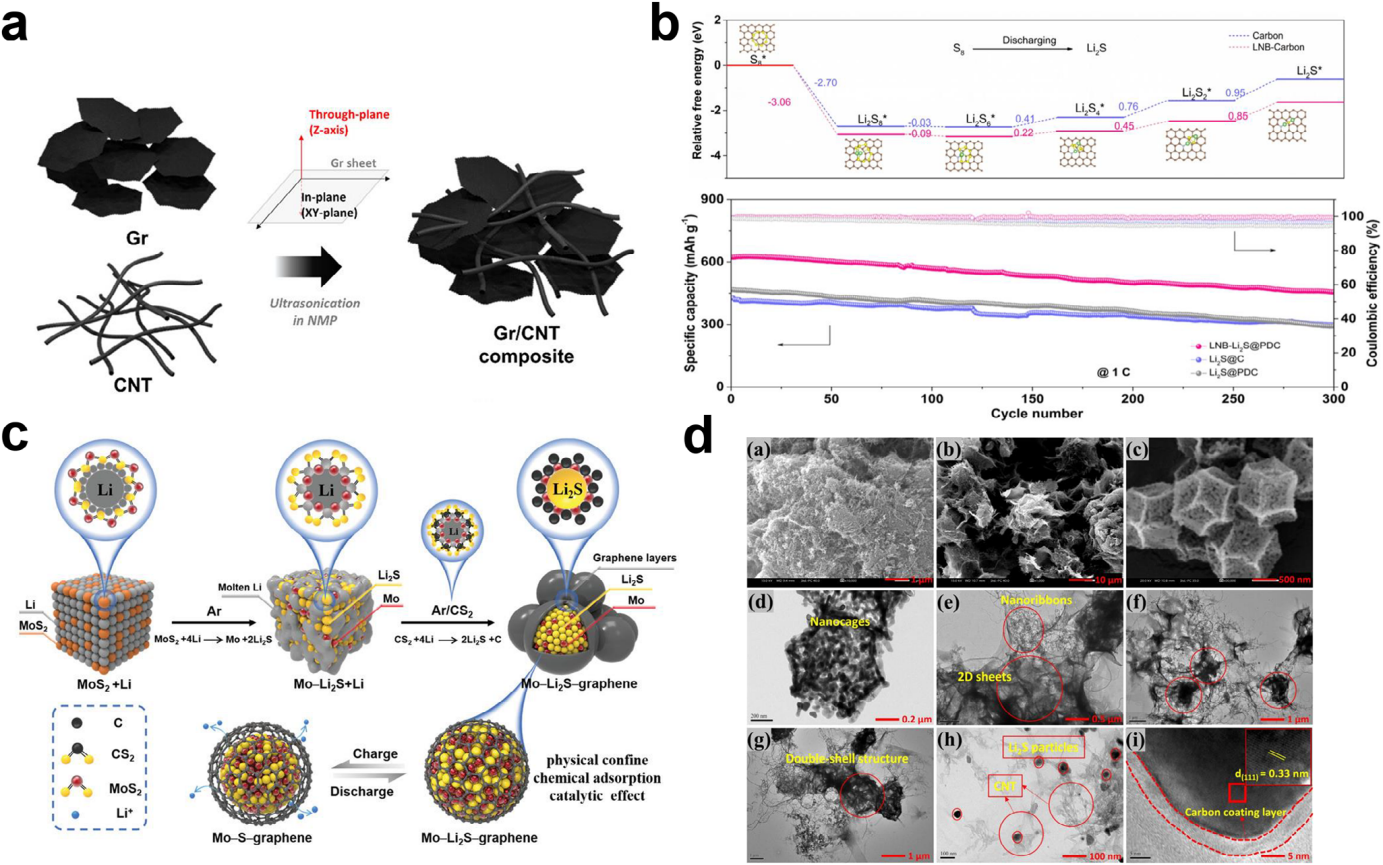
Different approaches to construct a sulfur‐carbon host. a) Schematic of the Graphene (Gr)/Carbon Nanotubes (CNT) composite preparation process. Reproduced under the terms of the CC‐BY 3.0 license [53]. Copyright 2019, Kim et al. b) Relative free energy profiles for the reduction of sulfur species on nanocarbon and Lithium‐Nitrogen Bond (LNB)‐nanocarbon. The long‐term cyclic performances of LNB‐ Li_2_S@PDC, Li_2_S@PDC and Li_2_S@C cathodes at 1C for 300 cycles. Reproduced with permission [27]. Copyright 2021, Elsevier. c) Schematic illustration of Mo‐Li_2_S‐graphene capsule architecture formed via a two‐stage lithiothermic reaction: MoS_2_ reacts with Li to generate Li_2_S─Mo nanocomponents, followed by excess Li reacting with CS_2_ to form Li_2_S─C shells that encapsulate the core. Reproduced with permission [33]. Copyright 2021, John Wiley and Sons. d) Scanning and Trasmission Electron Microscopies characterization of the Li_2_S─N─Co_3_O_4_/rGONR/CNT composite: SEM images (a–c) show 1D CNTs with abundant nanowires, 2D mesoporous rGONR sheets, and 3D porous N‐Co_3_O_4_ nanocages. Cross‐sectional SEM reveals a porous mixed carbon matrix beneficial for sulfur storage. TEM images d–i) confirm a well‐defined porous structure with Li_2_S particles encapsulated by an N─Co_3_O_4_/C double shell and a lattice spacing of 0.33 nm corresponding to the (111) plane of crystalline Li_2_S. Reproduced with permission [58]. Copyright 2024, American Chemical Society.

The promise of pairing Li_2_S cathodes with lithium‐free or silicon‐based anodes makes Li─S a next‐generation platform that combines high energy with the safety and manufacturability of Li‐ion technology [46]. Recent efforts have targeted the intrinsic hurdles, namely, the poor conductivity of Li_2_S and sluggish delithiation, through size control and composite design [35]. Although nanosizing lowers the first charge activation barrier, the most effective strategy to simultaneously enhance charge transport, confine polysulfides, and accelerate the interfacial kinetics is to embed Li_2_S nanoparticles within conductive carbon matrices (porous carbon, carbon nanotubes (CNTs), graphene, and carbon nanofibers), which are often coupled with electrocatalytic phases [47, 48, 49, 50, 51]. The carbon architecture and morphology are decisive. Pelletized Li_2_S/carbon aerogels delivered high utilization at practical loading (10 mg cm^−2^), retaining 955 mAh g^−1^ initially and 89% after 200 cycles; this performance extends to lithium‐freeTiO_2_/Li_2_S configurations [52]. Graphene–CNT networks compacted by high‐pressure milling, as showed in Figure 2a, achieved 5.3 mAh cm^−2^ at 0.2 C with > 800 cycles in graphite/Li_2_S full cells [53]. This produces high‐density cathodes with greatly improved particle–particle electronic contact and volumetric energy density; a canonical example compacts Li_2_S/graphene mixtures to achieve a >200% volume reduction. Furthermore, it has been successfully demonstrated in pouch‐type batteries using pelletized Li_2_S/graphene cathode material, clearly proving its practicality for scaling up to large‐scale lithium‐sulfur batteries [37, 53]. Metal‐free Li_2_S‐graphene composites paired with a biomass‐derived carbon anode achieved 805 mAh g^−1^ and maintained 340 mAh g^−1^ after 350 cycles without a high‐voltage activation step [54]. Beyond physical mixing, the chemical C─S bonds formed by lithiothermic routes stabilize the interfaces and improve their durability relative to that of their ball‐milled counterparts [55]. Additive manufacturing provides scalable pathways: Li_2_S/carbon electrodes 3D‐printed from Li_2_SO_4_ precursors exhibit 6.29 mAh cm^−2^ at 6 mA cm^−2^ (10 mg cm^−2^ loading) and simplify handling of air‐sensitive sulfides [56]. Host functionalization further strengthens polysulfide binding and catalysis. Heteroatom‐doped carbons (O, N, P, and S) improve active‐species anchoring and kinetics. Examples include r‐GONR/CNT scaffolds (≈500 mAh g^−1^, 71% retention, 200 cycles) [57] and N,S dual‐doped porous carbons (≈690 mAh g^−1^ at 1C, 85% retention, 200 cycles) [26]. Li─N catalytic motifs lower barriers for sulfide oxidation and aid Li^+^ transport (≈653 mAh g^−1^, 74.3% retention, 300 cycles), as demonstrated by the relative free energy profiles for the reduction of sulfur species on nanocarbon with Li─N bonds and by the electrochemical performance (see Figure 2b) [27]. Embedding transition metal catalysts, such as carbides, phosphides, oxides, or dual‐phase sulfides, results in strong, tunable interfacial activity. MoC‐based nanocarbons accelerate Li_2_S redox and bolster stability [27, 28]. CoFeP‐CN and NiMoP composites combine high conductivity with catalytic sites, yielding an approximately 991 mAh g^−1^ initial capacity and 0.029% fade per cycle over 800 cycles, which enabled anode‐free designs with approximately 50% retention over 300 cycles at a negative‐to‐positive capacity ratio (N/P) of 1 [30, 31, 32]. Co_9_S_8_/Co‐decorated Li_2_S in carbon hosts sustains > 1000 cycles at 1C (4.5 mg cm^−2^) and ≈ 653 mAh g^−1^ at 8.3 mg cm^−2^ (C/10) [32]. Lithiothermic metal‐Li_2_S‐graphene heterostructures (such as Mo–Li_2_S, Figure 2c) leverage spatial confinement and redox catalysis to retain 671.6 mAh g^−1^ after 500 cycles (3.5 mg cm^−2^) [33]. Ruthenium‐quantum‐dot catalysts decrease the Li_2_S decomposition barrier from 1.48 eV (carbon) to approximately 0.41 eV, enabling high rate capability and > 750‐cycle stability [34]. Polar oxide hosts (such as N─Co_3_O_4_/C) and VS_2_‐VO_2_ heterostructures chemisorb polysulfides and support practical loadings (≥ 3 mg cm^−2^) with sustained capacity (such as 1004 mAh g^−1^ at 0.1C; 413 mAh g^−1^ after 1000 cycles at 3C) [58, 59]. For example, the unique N─Co_3_O_4_/C‐based composite electrode exhibits significantly reduced internal resistance due to the 3D conductive pathways provided by the double‐shelled structure, as reported in Figure 2d. Moreover, the well‐interconnected micro‐/mesoporous architecture enhances Li^+^ diffusion and electron transport. In addition, the N─Co_3_O_4_ nanocages enable both physical and chemical confinement of polysulfides and act as electrocatalysts, markedly promoting Li_2_S conversion kinetics during the initial cycle [58].

### 2.1 Liquid Electrolytes: Mechanistic Role of Electrolytes

The electrochemical activation of Li_2_S in liquid‐electrolyte Li─S batteries is dictated by sluggish charge transfer and the large energy required to extract Li^+^ from its highly ionic, electronically insulating lattice. These coupled barriers produce the characteristic first‐charge overpotential of bulk Li_2_S, as Li^+^ removal must occur simultaneously with the nucleation and solvation of higher‐order polysulfides. Once oxidation begins, Li_2_S converts into soluble Li_2_S_x_ species (x > 2), initiating the familiar liquid‐phase redox sequence, while discharging reverses the process through polysulfide reduction and Li_2_S reprecipitation [60, 61, 62]. Because this conversion involves solid–liquid transitions and structural reorganization, the electrolyte is not a passive medium but an active participant that controls Li^+^ transport, polysulfide solubility, and interfacial kinetics. The solvation structure and donor strength of the electrolyte determine the desolvation energy at the Li_2_S surface and stability of the intermediate species. Therefore, appropriately formulated electrolytes can lower activation barriers, stabilize intermediates, and suppress the shuttle effect. Achieving a low overpotential, high sulfur utilization, and long‐term stability under lean electrolyte and realistic N/P conditions depends as much on the electrolyte chemistry as on the cathode architecture [63, 64]. Because performance under lean electrolyte and low N/P conditions is governed by tightly coupled electrolyte–electrode interactions and remains highly system‐specific, this Perspective emphasizes mechanistic dependencies and design targets rather than exhaustive performance benchmarking.

### 2.2 Materials and Electrolyte Co‐Design

**FIGURE 3:**
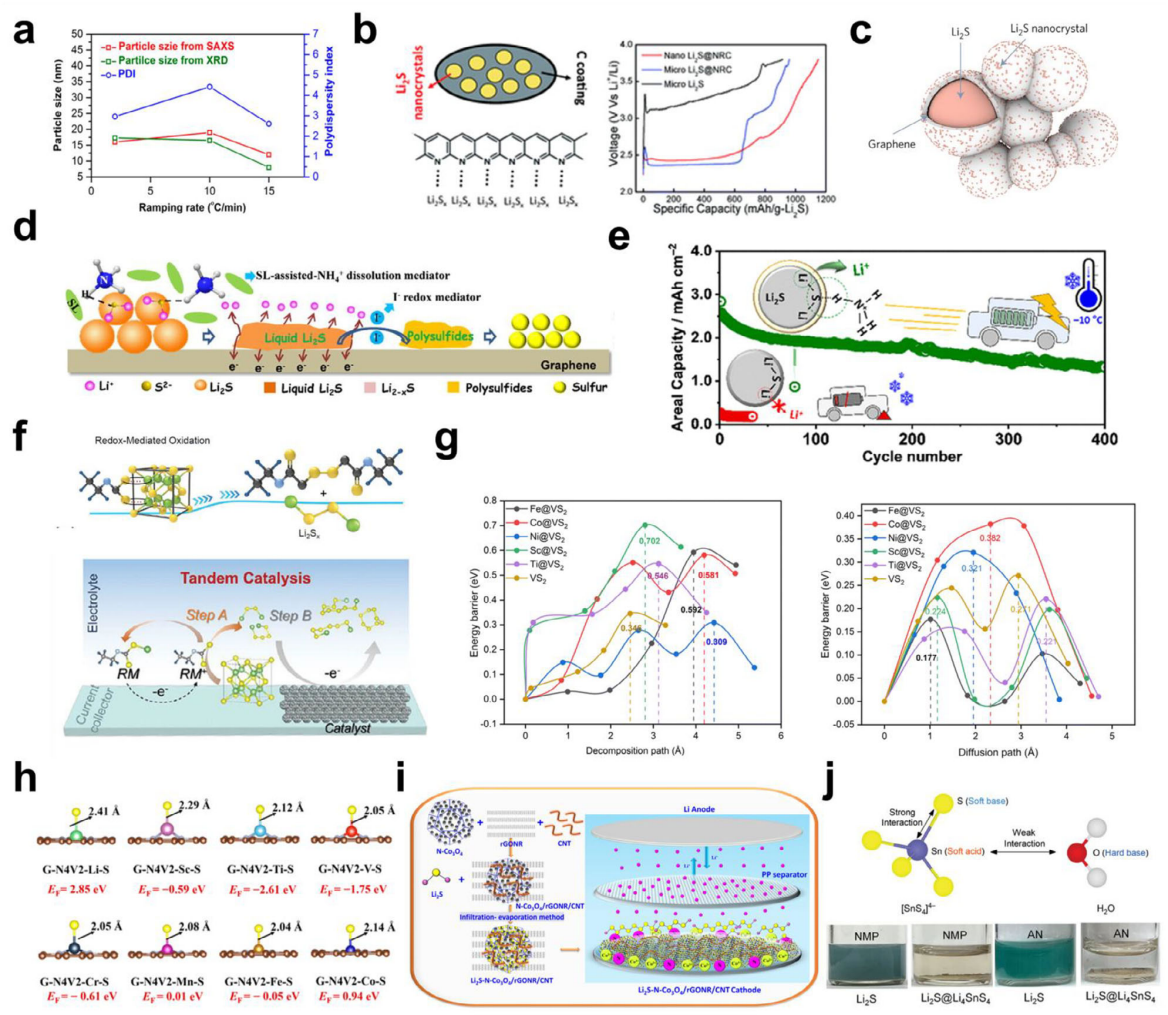
Strategies to overcome the limitations of Li_2_S materials. a) Comparison of the mean particle size (determined using X‐ray diffraction (XRD) and small‐angle X‐ray scattering (SAXS)) and polydispersity index of synthesized Li_2_S nano‐carbons (NCs) as a function of the ramp rate; Reproduced with permission [65]. Copyright 2019, American Chemical Society. b) Schematic illustrating nano‐Li_2_S@NRC composites and voltage profile of the first charge curves of Li_2_S with various grain sizes; Reproduced with permission [19]. Copyright 2013, Royal Society of Chemistry. c) Schematic illustrating Li_2_S nanocrystals encapsulated in graphene capsules; Reproduced with permission [67]. Copyright 2017, Springer Nature. d) Schematic diagram of the activation process of Li_2_S with a 0.5% SL‐NH_4_I additive in the standard electrolyte; Reproduced with permission [72]. Copyright 2020, Elsevier. e) Long‐term cycling of Li_2_S‐based Li‒S full batteries with a NH_4_NO_3_ additive at −10(C; Reproduced with permission [71]. Copyright 2023, American Chemical Society. f) Schematic illustrating the redox‐mediated Li_2_S oxidation and Li_2_S−LiEX cathode with a Ni−NC catalyst; Reproduced with permission [72]. Copyright 2024, John Wiley and Sons. g) DFT‐calculated decomposition barriers of Li_2_S and lithium diffusion barriers on VS_2_, Fe@VS_2_, Co@VS_2_, Ni@VS_2_, Ti@VS_2_, and Sc@VS_2_; Reproduced with permission [80]. Copyright 1999, Royal Society of Chemistry. h) Adsorption energy of single sulfur atom M─N_4_‐based SACs (M = Sc (red lilac), Ti (blue lilac), V (red), Cr (steel blue), Mn (traffic purple), Fe (broom yellow), and Co (blue)). Brown color represents C, yellow represents S, and light blue represents N; Reproduced under terms of the CC‐BY‐NC‐ND license [79]. Copyright 2023, The Authors, published by American Chemical Society. i) Schematic illustrating the Li_2_S─N─Co_3_O_4_/rGONR/CNT composite; Reproduced with permission [58]. Copyright 2024, American Chemical Society. j) Design principle of an air‐stable Li_2_S composite based on the hard–soft acid–base principle and reactivity test of Li_2_S and the Li_2_S@Li_4_SnS_4_ composite in N‐methyl‐2‐pirrolidone (NMP) and acetonitrile (AN) solvents; Reproduced with permission [86]. Copyright 2024, John Wiley and Sons.

The most effective strategies for enhancing Li_2_S performance mandate the coupled design of the cathode and electrolyte. Figure 3 summarizes the strategies to overcome the limitations of Li_2_S materials. At the materials level, nanoscale Li_2_S particles embedded within conductive matrices, such as porous carbon, CNTs, or graphene, minimize diffusion paths and enhance electron transport (Figure 3a–c) [19, 65, 66, 67]. Heteroatom doping (N, S, O, and P) transforms carbon from a passive conductor into an active host capable of polar binding and the catalytic conversion of polysulfides via Lewis acid–base and electrostatic interactions [21, 26, 51, 68]. Porosity engineering and 3D frameworks allow high areal loading with low tortuosity and effective ion/electron accessibility, thereby improving utilization and cycle stability. Solvation tuning plays an equally decisive role on the electrolyte side. Localized high‐concentration electrolytes (LHCEs) and weakly solvating cosolvents suppress polysulfide dissolution while retaining ionic conductivity. Bifunctional additives such as ethanol or ammonium salts partially dissolve Li_2_S and facilitate solid–liquid transformation, reducing activation voltage. For example, trace ethanol transforms the Li_2_S interface into a mixed solid–liquid region that enhances Li^+^ mobility and electronic contact, while 0.25 m NH_4_NO_3_ in dimethoxyethane, (DME): 1,3‐dioxolane (DOL) produces transient liquid‐phase Li_2_S via NH_4_^+^‒S^2−^ interactions, enabling activation even at –10°C and long‐term cycling (Figure 3d,e) [69, 70, 71, 72].

Redox mediators (RMs) are alternative chemical activation methods. Electrochemically generated species such as lithium ethyl xanthate (LiEX; ≈ 2.3 V vs Li^+^/Li) oxidize Li_2_S to soluble polysulfides with minimal energetic penalty. Combined with a Ni─N─C catalyst, this strategy lowers the Li_2_S oxidation plateau from ≈ 3.6 to ≈ 2.3 V and delivers > 1000 mAh g^−1^ with an extended cycle life (Figure 3f) [73]. Transition‐metal catalyst oxides, sulfides, phosphides, and carbides accelerate interfacial reactions by providing accessible *d*‐states that hybridize with S *p*‐orbitals, facilitating inner‐sphere electron transfer and lowering the activation barriers (Figure 3g) [30, 74, 75, 76, 77, 78, 79, 80].

Single‐atom catalysts (M–N_x_ or M–S_x_) maximize atomic efficiency and offer precisely tunable coordination for selective polysulfide activation, while DFT and operando spectroscopy now enable rational screening of active sites (Figure 3h) [79, 80]. At the device level, catalytic interlayers and modified separators extend these effects by spatially confining the soluble species and catalyzing their reconversion. Concurrently, polar or catalytic interlayer‐MXenes, nitrides, or oxide/nitride heterostructure‐bridge cathode and separator interfaces spatially confine soluble species while sustaining electron and ion transport. Mechanistically, these multifunctional layers stabilize sulfur intermediates in polar domains, while adjacent conductive subphases accelerate redox conversion, enhancing the Coulombic efficiency and lifespan (Figure 3i) [58, 81, 82, 83]. Finally, because Li_2_S is highly reactive toward moisture and oxygen, practical processing demands air‐stable protection. Polymer or inorganic sheaths that block ambient exposure but later transform into ionically conductive interphases simultaneously preserve the stability and electrochemical access. The Li_2_S and Li_2_S/SnS_2_ solutions turned blue due to Li_2_S dissolution in NMP and AN, whereas Li_2_S@Li_4_SnS_4_ remained colorless, indicating effective encapsulation. The Li_4_SnS_4_ shell thus shields Li_2_S from moisture and suppresses polysulfide leakage (Figure 3j) [84, 85, 86].

### 2.3 Toward Practical Full Cells

**FIGURE 4:**
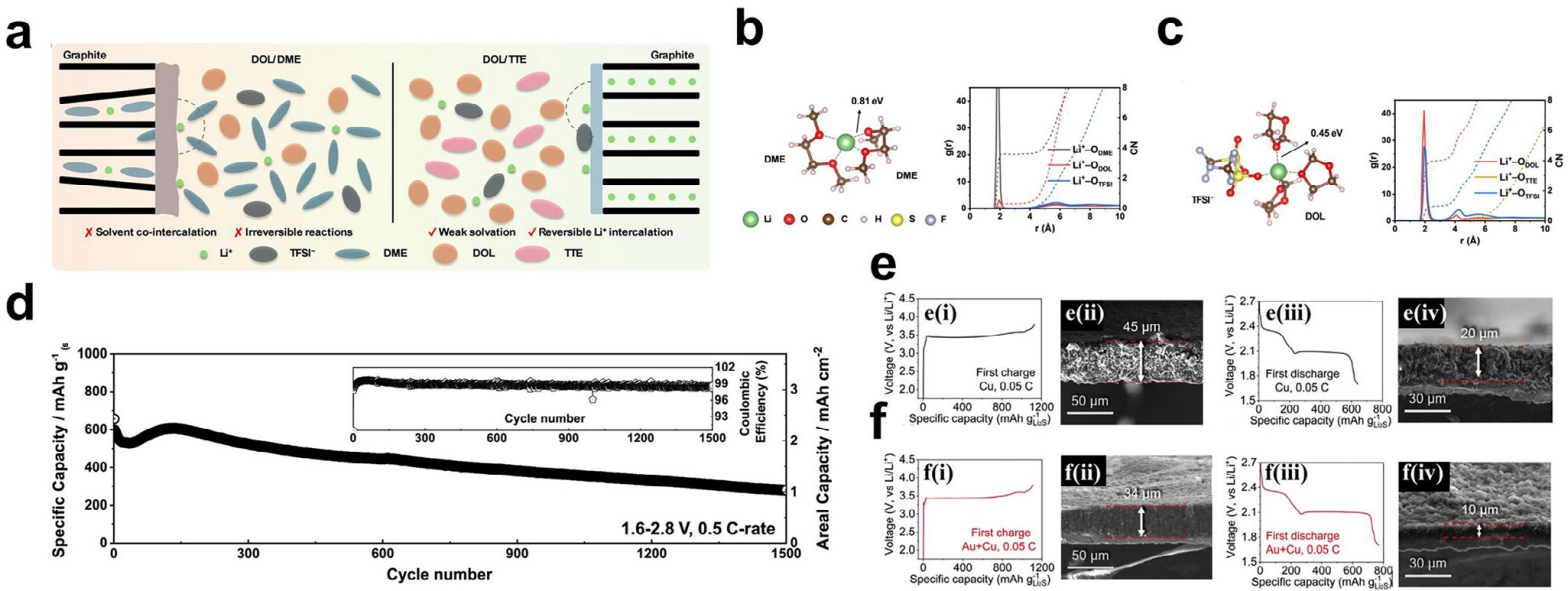
a) Schematic of solvation structure and intercalation behaviors of DOL/DME and DOL/ 1,1,2,2‐Tetrafluoroethyl‐2,2,3,3‐tetrafluoropropylether (TTE) electrolytes with graphite anodes. The radial distribution function (RDF) and coordination number derived from the MD results for the b) DOL/DME and c) DOL/TTE electrolyte; Reproduced with permission [83]. Copyright 2014, Royal Society of Chemistry. d) Long‐term cycling test of a 2D‐LGW cathode and graphite anode; Reproduced with permission [76]. Copyright 2020, American Chemical Society. Galvanostatic charge–discharge profiles and the corresponding SEM images of e) the Li_2_S||Cu and f) Li_2_S||Au/Cu cells for the first cycle at 0.05C; Reproduced with permission [84]. Copyright 2020, Elsevier.

The transition from half‐cell demonstrations to practical full batteries requires the coordinated optimization of the cathode, electrolyte, and anode interfaces. In graphite/Li_2_S configurations, the electrolyte must prevent solvent co‐intercalation, suppress polysulfide shuttling, and form a robust, LiF‐rich solid electrolyte intephase (SEI) that remains stable in the presence of Li_2_S‐derived species [87, 88]. Solvate ionic liquids and fluorinated co‐solvents have proven effective in tuning the solvation and interfacial chemistry, enabling long‐life graphite‐Li_2_S cells with high reversibility (Figure 4a–d) [82, 88, 89]. In addition to graphite, anode‐ or Li‐free architectures offer enhanced safety and simplified manufacturing. The electrolyte composition and current‐collector design in anode‐ or Li‐free architectures govern the Li nucleation and stripping behavior. Gold‐modified copper foils and 3D lithiophilic scaffolds reduce the nucleation barriers and produce uniform, dendrite‐free lithium deposits, while alloy‐forming seed layers, such as gold, silver, or nickel, maintain the interfacial stability. After the first charge to 3.8 V, Li deposition on Au/Cu is thinner and more uniform than on bare Cu despite similar initial charge behavior. The Au/Cu electrode retains much less residual Li after discharge and delivers higher capacity, indicating more efficient and reversible Li utilization (Figure 4e,f) [90, 91, 92, 93, 94]. Li_2_S@Ni_x_MoᵧP_z@C cathodes paired with thin Ni collectors recently delivered stable cycling for hundreds of cycles at N/P ≈ 1, underscoring the promise of integrated catalyst–collector designs [31]. Quasi‐solid and gel electrolytes that retain the coordination chemistry of LHCEs while suppressing polysulfide shuttling further enhance safety and cycle life, bridging liquid and solid‐state operation [32, 95, 96, 97, 98]. Collectively, these developments illustrate that the electrolyte design now dictates the direction of Li─S chemistry. By mediating Li_2_S redox through tailored solvation, catalytic additives, and stable interfaces, next‐generation liquid systems are evolving from reactive solvents to integrated electrochemical frameworks capable of S1 lithium‐free, safe, and high‐energy Li_2_S batteries. A summary of these findings is reported in Table 1.

**TABLE 1:** Conceptual comparison of anode options paired with Li_2_S cathodes in Li─S batteries [44, 87, 88, 89, 90, 91, 92, 93].

Anode type	Interface stability with Li_2_S derived species	Volume change	Cycle‐life implications	Key advantages	Key limitations
Graphite	Moderate–high (stable SEI possible with fluorinated/LHCE electrolytes; limited polysulfide reactivity)	Low (∼10%)	Long cycle life (>300–500 cycles demonstrated)	Mature manufacturing, low swelling, compatibility with Li_2_S activation below 2.5 V	low swelling, compatibility with Li_2_S activation below 2.5 V Risk of solvent co‐intercalation; requires electrolyte engineering
Silicon/SiOx	Moderate (sensitive to polysulfides; requires protective coatings or solid electrolytes)	Very high (∼300–400%)	Limited unless mechanically buffered	High specific capacity; enables Li‐metal‐free architectures	Severe mechanical stress; interface instability
Lithium metal	Poor intrinsic stability (reacts with polysulfides and liquid electrolytes)	Infinite (plating/stripping)	Short cycle life unless protected	Highest energy density; simplest kinetics	Dendrites, safety risk, poor manufacturability
Anode‐free (Cu based)	Interface defined by Li nucleation efficiency	No host expansion	Cycle life limited by Coulombic efficiency	Simplified design; high volumetric energy density	Requires >99.9% plating efficiency

## 3 Solid‐State Electrolytes (SSEs): Electrochemical Mechanism of ASSLSBs

**FIGURE 5:**
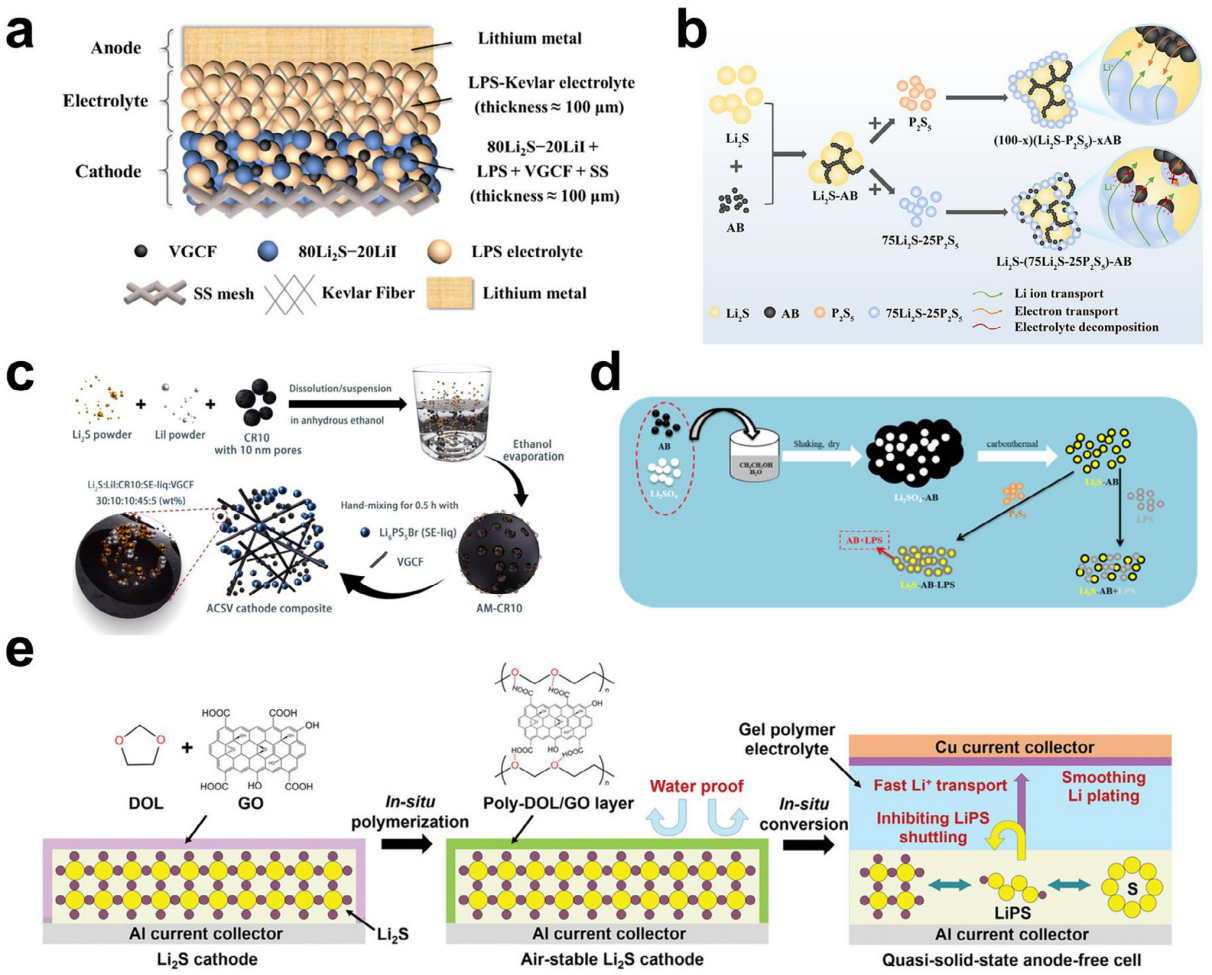
Electrochemical mechanism in ASSLSBs. a) Schematic illustrating the architecture of ASSLSBs; Reproduced with permission [99]. Copyright 2019, American Chemical Society. b) Fabrication pathway for solid‐state sulfur cathode composites; Reproduced with permission [100]. Copyright 2023, American Chemical Society. c) Schematic illustrating the synthesis process for the sulfur cathode composite material; Reproduced under terms of the CC‐BY license [108]. Copyright 2024, The Authors, published by Springer Nature. d) Schematic depicting the Li_2_S–AB–LPS and Li_2_S–AB/LPS‐type cathode fabrication process; Reproduced with permission [103]. Copyright 2021, Elsevier. e) Schematic illustrating the fabrication of a Li_2_S cathode via the in situ polymerization of DOL and GO; Reproduced with permission [97]. Copyright 2022, John Wiley and Sons.

Replacing liquid electrolytes with solid‐state ion conductors fundamentally reshapes Li─S chemistry by eliminating polysulfide shuttling, improving safety, and enabling high‐voltage operation. All‐solid‐state Li─S batteries (ASSLSBs) address the core limitations of liquid systems, including insulating active materials, ∼80% volume expansion, and interfacial degradation, by converting the sulfur redox reaction into a solid–solid reaction mediated by solid electrolytes. Unlike conventional liquid‐electrolyte Li─S batteries, ASSLSBs fundamentally redefine the reaction pathway by eliminating the liquid phase and associated solid–liquid intermediates. This transformation suppresses polysulfide dissolution and diffusion, as the redox process proceeds entirely through solid–solid conversion, that is, sulfur directly reacts with Li^+^ ions conducted through the solid electrolyte to form Li_2_S and does not generate soluble polysulfides. Recent study have notably identified a solid‐state sulfur redox mechanism in which the electrolyte participates catalytically [98]. Lithium thioborophosphate iodide (LBPSI) acts not only as a fast lithium‐ion conductor but also as a surface RM. During charging, interfacial I^−^ is electrochemically oxidized to I_2_/I_3_^−^, which subsequently oxidizes Li_2_S at the Li_2_S/electrolyte boundary. This two‐phase boundary activation, which is considerably more abundant than the conventional three‐phase contact of liquid systems, markedly accelerates the reaction kinetics and enhances the utilization of active sites. Figure 5 summarizes the representative structural and mechanistic advances in solid‐state Li─S electrochemistry. Figure 5a schematically illustrates the typical architectural configuration of an ASSLSB, which is comprised of a lithium‐metal anode, sulfide solid electrolyte, and Li_2_S–carbon composite cathode, where close interfacial contact ensures continuous Li^+^/e^−^ pathways [99]. Figure 5b,c schematically illustrate the synthetic routes for solid‐state sulfur cathodes, showing how nanoscale sulfur or Li_2_S species are uniformly incorporated into conductive carbon frameworks or derived from molecular precursors, thus maximizing electrochemically active interfaces [100, 101].

Figure 5d schematically illustrates the fabrication of Li_2_S–AB–LPS composites, demonstrating how the intimate integration of acetylene black and a Li_6_PS_5_Cl electrolyte yields a bicontinuous ion/electron conduction network, essential for fast redox conversion [49]. Finally, Figure 5e shows an in situ polymerization strategy in which DOL reacts with graphene oxide (GO) to form a gel polymer matrix that stabilizes the Li_2_S interface, improves Li^+^ transport, and suppresses polysulfide migration [102]. Collectively, these designs exemplify how interfacial engineering and hybrid composite architectures demonstrate a path for transitioning the conventional liquid‐mediated Li─S chemistry to a fully solid‐state, diffusion‐limited redox system that offers superior kinetics and stability.

### 3.1 SSEs for Li‒S Batteries

**FIGURE 6:**
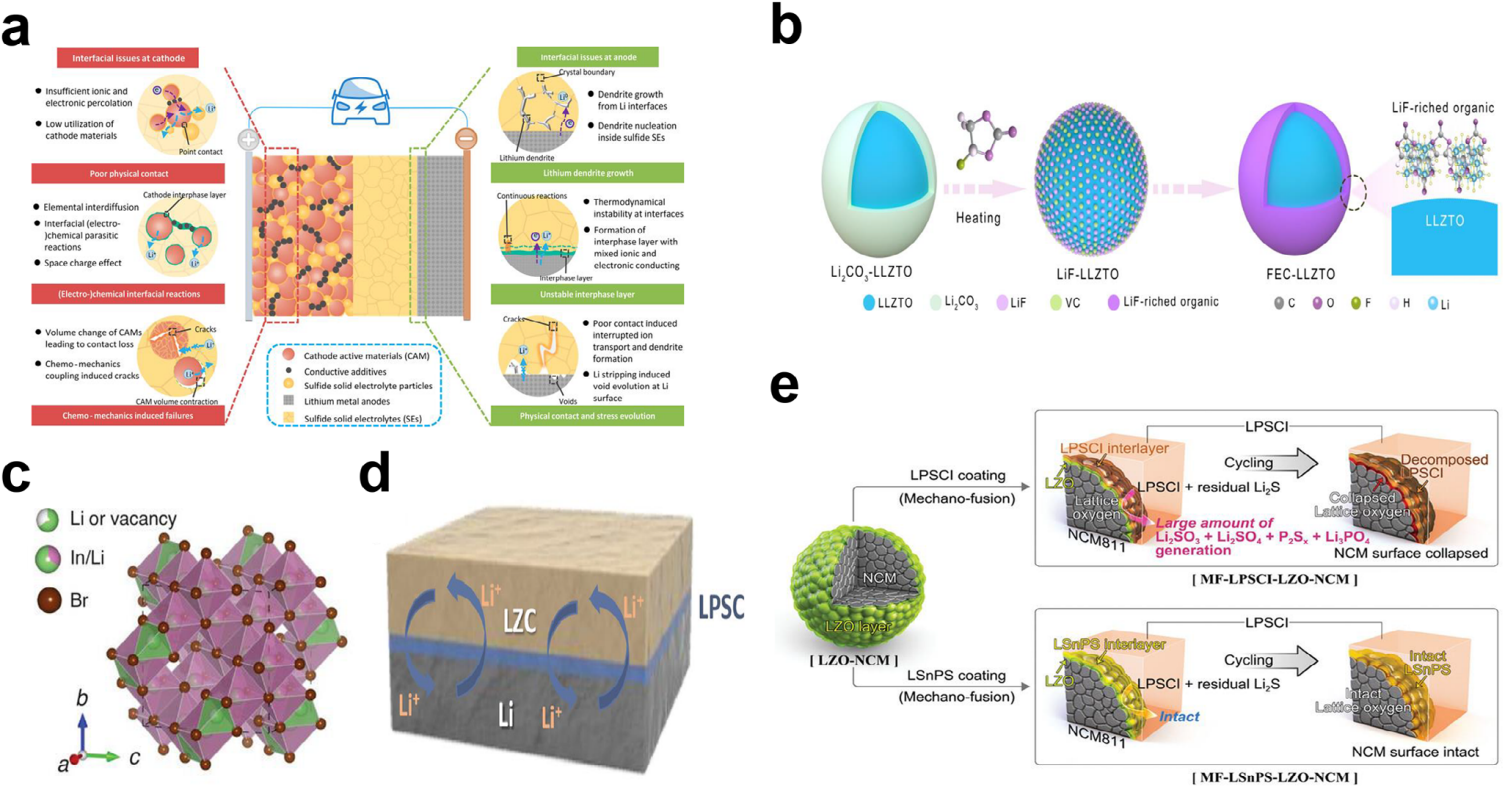
Interface and structural characteristics of solid electrolytes in all‐solid‐state lithium‐ion batteries. a) Schematic illustration of various interfacial issues in all‐solid‐state lithium‐ion batteries based on sulfide solid electrolytes; Reproduced under the terms of the CC‐BY license [104]. Copyright 2022, Liang et al. b) Schematic diagram of the preparation mechanism for fluoroethylene carbonate (FEC)‐modified lithium lanthanum zirconium titanium oxide (LLZTO); Reproduced with permission [107]. Copyright 2025, Elsevier. c) Refined crystal structure diagram of powdered Li_3_InBr_6_; Reproduced under the terms of the CC‐BY‐NC‐ND license [109]. Copyright 2025, Fu et al. d) Schematic of the contact interface between sulfide electrolyte (LPSC)‐lithium lanthanum zirconium oxide (LLZO) composite solid electrolyte and lithium anode; Reproduced with permission [110]. Copyright 2023, Elsevier. e) Schematic diagrams of surface structures and cycling degradation mechanisms for modified sulfide electrolyte (MF‐LPSCI, MF‐L_8_SnPS_10_) and NCM cathode composite system (LZO‐NCM); Reproduced with permission [111]. Copyright 2023, John Wiley and Sons.

SSEs are critical in ASSLSBs because they dictate both Li^+^ transport and electrode‐electrolyte compatibility. Four major classes of SSE materials have been developed, namely, sulfide‐, oxide‐, halide‐, and polymer‐based materials, each with distinct advantages and disadvantages. Sulfide electrolytes are among the most promising for ASSLSBs owing to their ultrahigh ionic conductivities (10^−3^–10^−2^ S cm^−1^ at room temperature) and excellent mechanical ductility. Their plasticity allows intimate electrode contact through cold pressing, minimizing the interfacial resistance without high‐temperature sintering [103]. Representative examples include Li_7_P_3_S_11_, Li_6_PS_5_X (X = Cl, Br, and I), and lithium thioborophosphate iodide (LBPSI) glass‐ceramics. Figure 6a schematically illustrates multidimensional interfacial challenges in sulfide‐based all‐solid‐state lithium‐ion batteries, encompassing cathode‐side issues (insufficient ion/electron transport, parasitic reactions, chemomechanical failure) and anode‐side challenges (dendrite growth, interfacial instability, contact loss), alongside corresponding evolution of electrode‐electrolyte interface structures [104]. Notably, Song et al. [98] demonstrated that LBPSI enables ultrafast charging (150C at 60°C) and ultralong life (25 000 cycles with 80.2% retention) through a reversible I^−^/I_2_/I_3_^−^ redox process that accelerates Li_2_S activation. Oxide electrolytes, characterized by wide electrochemical windows (≈0–5 V vs. Li/Li^+^) and excellent thermal stabilities, are attractive candidates for high‐voltage ASSLSBs. The most studied member, Li_7_La_3_Zr_2_O_12_ (LLZO), offers an ionic conductivity of approximately 10^−4^ S cm^−1^ at room temperature [105]. Modified compositions such as Li_6.25_Al_0.25_La_3_Zr_2_O_12_ further improve stability and lithium‐metal compatibility, effectively protecting Li from corrosion and ensuring sustained ion migration in solid conductors [106]. On the other hand, Figure 6b demonstrates that LLZO containing Li_2_CO_3_ impurities can form a Li F‐rich organic protective layer on LZTO after FEC heat treatment, eliminating surface impurities and enhancing interface compatibility [107]. Halide electrolytes have recently emerged as promising candidates for high‐voltage ASSLSBs owing to their chemical stability with Ni‐rich cathodes (such as LiNi_0.8_Co_0.1_Mn_0.1_O_2_) and relatively low sintering temperatures. Prototypical halides such as Li_3_YCl_6_, Li_2_ZrCl_6_, and LiScCl_4_ exhibit ionic conductivities of 10^−5^–10^−4^ S cm^−1^ at room temperature. Hakarid et al. [108] demonstrated that the discharge capacity of Li_2_S─Li─halide‐C composite ASSBs correlates with the halide ionic conductivity, with the order LiI > LiBr > LiCl, confirming transport‐limited behavior. As an example, Figure 6c presents the refined crystal structure of Li_3_InBr_6_ solid electrolyte, revealing the migration pathway of Li^+^ through Li vacancies/interstitial sites within the In‐Br framework [109]. Figure 6d illustrates the surface structure and cycling degradation mechanisms after coating LZO‐modified NCM cathodes with modified sulfide electrolytes (LPSCl, LSnPS). where LZO‐modified NCM exhibits severe interface collapse and decomposition into Li_2_S, Li_3_PO_4_, etc., after cycling with LPSCl coating. In contrast, LSnPS with superior oxidation stability maintains intact NCM surface structure with negligible decomposition products after extended cycling, indicating the formation of an electrochemically stable NCM‐LSnPS interface [110]. Finally, Figure 6e demonstrates the interface between LPSC‐LLZO composite solid electrolyte (LZC), combining the two types of solid electrolytes, and lithium anodes, where its biphasic composite structure promotes uniform Li^+^ transport and deposition while suppressing lithium dendrite growth [111]. Polymer electrolytes bridge the gap between liquid and solid systems, offering flexibility and safety in anode‐free configurations. Polyethylene oxide matrices complexed with lithium salts such as lithium bis(trifluoromethanesulfonyl)imide (LiTFSI) support Li^+^ conduction while suppressing polysulfide migration and eliminating flammable solvents. The performance can be enhanced by incorporating inorganic fillers (such as LLZO) or ionic liquids, thereby improving both the conductivity and interfacial wettability [102]. Recent studies also integrated RMs within polymer matrices. Xin GAO et al. [112] reported that anthraquinone (AQT) catalyzes Li_2_S oxidation with excellent potential alignment and reversibility, with Li_2_S@AQT cells yielding a 98.9% Coulombic efficiency after 150 cycles and exhibiting a robust rate performance.

### 3.2 Key Challenges of ASSLSBs

**FIGURE 7:**
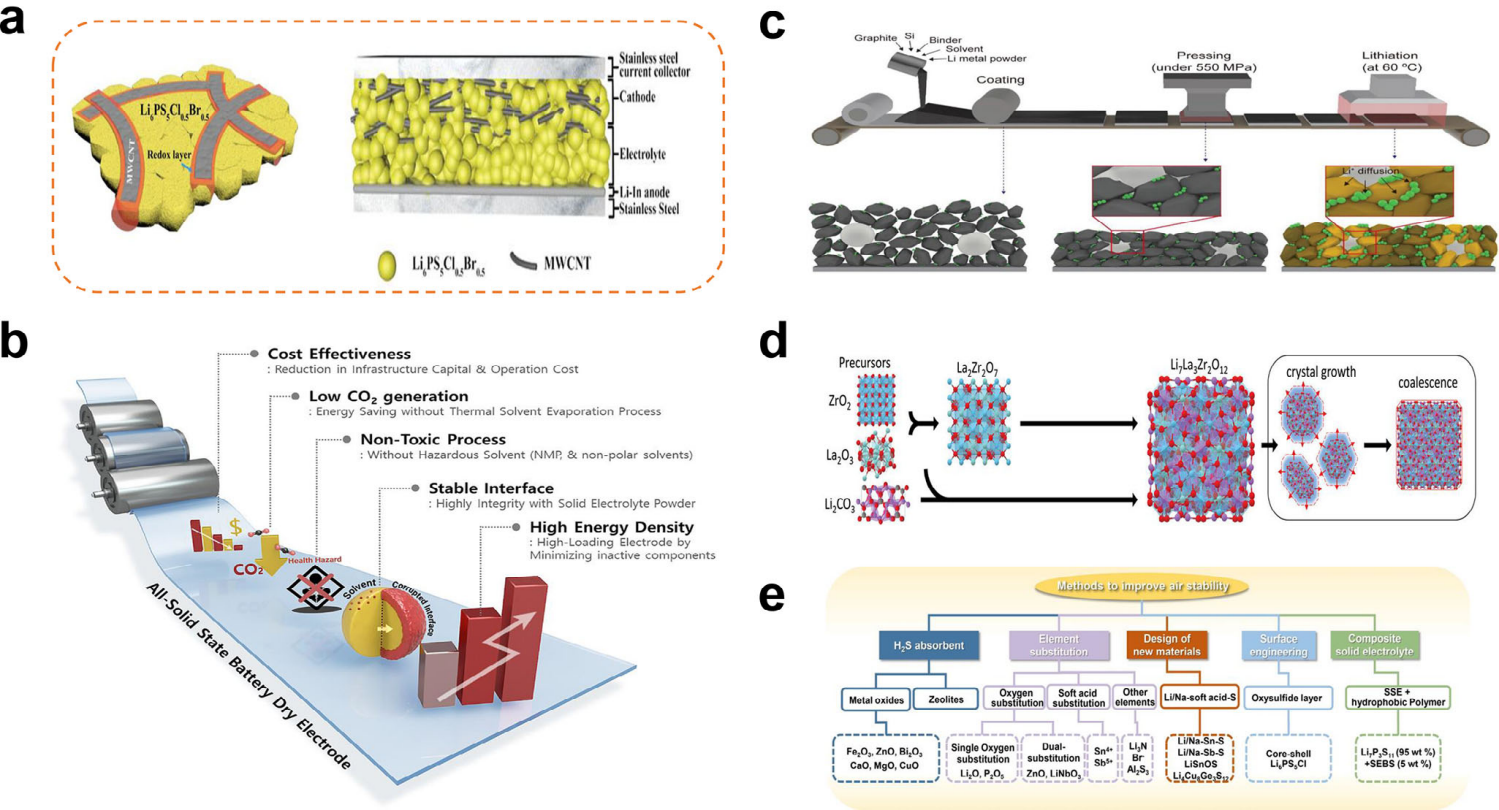
Schematic diagram of key structures, fabrication processes, and performance optimization strategies for all‐solid‐state lithium‐sulfur batteries. a) Schematic diagram of the LPSCB‐MWCNTs composite electrode and the In/InLi | LPSCB | LPSCB‐MWCNTs full cell structure.The black, orange, and yellow regions correspond to MWCNTs, the redox layer, and LPSCB, respectively; Reproduced under the terms of the CC‐BY‐NC‐ND license [114]. Copyright 2021, Wang et al. b) Schematic Diagram of Advantages of Dry‐Processed Electrodes for All‐Solid‐State Batteries; Reproduced under the terms of the CC‐BY‐NC license [115]. Copyright 2025, Mun et al. c) Illustration of the dry‐process electrode fabrication workflow; Reproduced under the terms of the CC‐BY‐NC license [116]. Copyright 2023, Lee et al. d) Synthetic pathway and growth schematic for LLZO solid‐state electrolyte precursors; Reproduced under the terms of the CC‐BY‐NC license [117]. Copyright 2023, Zheng et al. e) Taxonomy of optimization methods for solid‐state electrolyte air stability; Reproduced with permission [118]. Copyright 2022, Springer Nature.

Although ASSLSBs promise exceptional safety, longevity, and a theoretical energy density approaching 2600 Wh kg^−1^, their practical realization remains constrained by several intrinsic scientific and engineering challenges. A primary limitation arises from the extremely low electronic (< 10^−14^ S cm^−1^) and ionic conductivities of both elemental sulfur and its discharge product, Li_2_S, which lead to incomplete active material utilization and sluggish redox kinetics [113]. Although lithium‐sulfur‐silver‐germanium‐type electrolytes (e.g., Li_6_PS_5_Cl) can serve as cathode precursors to enhance interfacial conductivity, their compatibility with sulfur‐based active materials still requires optimization [114]. These transport limitations become even more severe in solid‐state architectures, where reactions are restricted to localized solid–solid contact regions instead of the more continuous three‐phase boundaries (electrolyte/active material/conductive additive) found in liquid systems. Additionally, the approximately 80% volume change accompanying the reversible S ↔ Li_2_S conversion imposes large mechanical stresses that can crack electrodes, disrupt interfacial contact, and accelerate capacity fading, especially in high‐loading cathodes designed for practical energy densities [49]. Furthermore, the loss of ion conduction pathways at the Li_2_S‐electrolyte interface disrupts reaction continuity and rate capability, hindering high‐power battery output. Dry electrode technology offers a viable solution to mitigate these challenges. A sulfur cathode fabricated using a polytetrafluoroethylene (PTFE) fiber‐forming process utilizes a fiber network to buffer expansion stresses, maintaining structural integrity even at a sulfur loading of 4.5 mg·cm^−2^. Low PTFE content (0.1–1 wt.%) further reduces ionic conduction hindrance [115]. Concurrently, dry pre‐lithiation strategies introduce pre‐lithiation agents (e.g., metallic lithium powder) during electrode fabrication. This approach compensates for lithium depletion during initial cycling of sulfur cathodes and indirectly mitigates interfacial delamination caused by volumetric changes [116]. The resulting loss of ionic percolation at the Li_2_S‐electrolyte interface severely hampers reaction continuity and rate capability. At the anode side, thermodynamic instability between sulfide electrolytes (such as Li_7_P_3_S_11_) and metallic lithium leads to interfacial decomposition, producing resistive phases such as Li_3_P and Li_2_S that increase impedance and promote uneven lithium deposition [44]. Furthermore, surface modification studies on oxide electrolytes (e.g., Li_7_La_3_Zr_2_O_12_, LLZO) provide insights for cross‐system interface optimisation. In situ heating to regulate the amorphous degree of the LLZO surface reduces its reactive interface with metallic lithium [117]. Finally, air and moisture sensitivities remain major barriers to scalable fabrication [118]. Both Li_2_S and thiophosphate‐based SSEs readily hydrolyze in humid environments, generating toxic H_2_S gas and degrading electrochemical performance. This necessitates rigorous handling and encapsulation in an inert atmosphere, which complicates manufacturing and increases production costs [119]. Researchers are enhancing the storage stability and safety of battery components by designing and applying artificial protective layers, including hydrophobic coatings and chemically inert coatings, to shield them from atmospheric corrosion [84, 120, 121]. Figure 7 presents some of the challenging and solutions discussed above. Figure 7a shows the structure of Li_6_PS_5_Cl_0.45_Br_0.55_ electrolyte particles with MWCNT conductive network and coating layer on the left, and the battery stack structure on the right, which consists of stainless steel current collector, cathode, electrolyte, Li‐In anode, and stainless steel substrate in sequence [114]. Figure 7b highlights the advantages of dry‐process electrodes for all‐solid‐state batteries, including cost‐effectiveness, environmental friendliness, interfacial stability, and high energy density [115]. Figure 7c depicts the preparation process of the dry electrode, from left to right: coating of graphite, sulfur, binder, and electrolyte powders; roll compaction at 550 MPa; and lithiation treatment at 60°C, along with corresponding microstructural changes at each step [116]. Figure 7d illustrates the growth process of LLZO crystals using ZrO_2_, La_2_O_3_, and Li_2_CO_3_ as precursors, involving composite formation, crystallization, growth, and agglomeration [117]. Figure 7e presents optimization methods for enhancing the air stability of solid‐state electrolytes, encompassing strategies such as H_2_S adsorbents (metal oxides, zeolites, etc.), elemental doping, novel oxide design, surface modification, and composite reinforcement, alongside representative material systems corresponding to each strategy [119].

### 3.3 Material Design and Electrode Engineering

**FIGURE 8:**
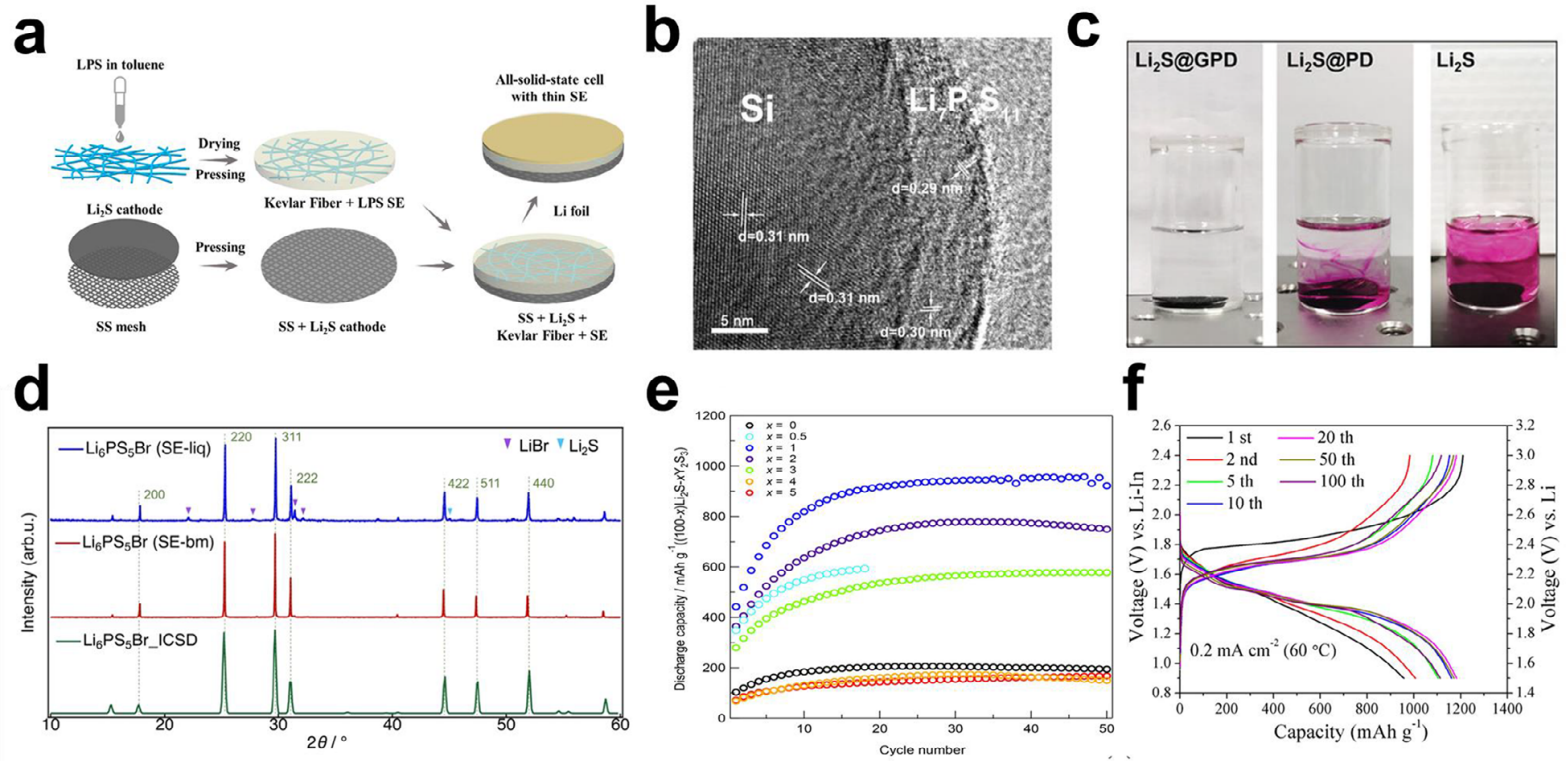
Materials design and electrode engineering for ASSLSBs. a) Schematic of the fabrication of a supporting cathode with a thin sulfide SSE; Reproduced with permission [100]. Copyright 2019, American Chemical Society. b) High‐resolution transmission electron microscopy (HRTEM) image of the Si@LPS composite; Reproduced with permission [44]. Copyright 2019, Springer Nature. c) Optical images showing aqueous phenolphthalein indicator solutions in contact with bare Li_2_S, Li_2_S@PD, and Li_2_S@GPD cathodes; Reproduced with permission [102]. Copyright 2022, John Wiley and Sons. d) XRD patterns of the Li_6_PS_5_Br SSEs synthesized using the liquid‐phase and ball‐milling methods; Reproduced with permission [125]. Copyright 2024, Springer Nature. Reproduced under terms of the CC‐BY license [125]. Copyright 2024, The Authors, published by Springer Nature. e) Cycling performance of the (100 – *x*)Li_2_S·*x*Y_2_S_3_ active cathode materials (*x*  = 0, 0.5, 1, 2, 3, 4, and 5) at 0.05C; Reproduced with permission [101]. Copyright 2023, American Chemical Society. f) Galvanostatic charge–discharge voltage profiles of an ASSLSB assembled with a Li_2_S@C nanocomposite cathode evaluated at 60°C under an areal loading of approximately 1.75 mg cm^−2^; Reproduced with permission [49]. Copyright 2019, American Chemical Society.

Addressing the intrinsic challenges of ASSLSBs, which include the insulating nature of sulfur and Li_2_S, substantial volume changes, and unstable solid–solid interfaces, requires coordinated advances in material design and electrode engineering. These efforts have focused on improving ionic and electronic transport, stabilizing interfaces, and supporting the high areal loading of active materials. Strategies for sulfur cathodes primarily focus on enhanced conductivity, interfacial kinetics, and structural resilience. Integrating active sulfur species with conductive carbon scaffolds is one of the most effective methods for overcoming electronic insulation. For instance, an in situ‐formed Li_2_S@C nanocomposite, synthesized via the combustion of lithium metal with CS_2_, uniformly embedded Li_2_S nanocrystals (50–100 nm) within a carbon matrix. This architecture shortened Li^+^ and electron diffusion paths, enabling 91% Li_2_S utilization at a loading of 7 mg cm^−2^, corresponding to an areal capacity of 7.46 mAh cm^−2^ [49]. Similarly, a Li_2_S–acetylene black composite prepared by the carbothermal reduction of Li_2_SO_4_ at 800°C exhibited a five‐order‐of‐magnitude enhancement in electronic conductivity, as compared to pristine Li_2_S, and achieved 73.9% utilization [113]. Halogen doping provides an alternative approach for improving ionic transport and redox activity. Doping with AlI_3_ via planetary ball milling increased the ionic conductivity of Li_2_S to 6.0 × 10^−5^ S cm^−1^, which is three orders of magnitude higher than that of the undoped material, and a capacity of 936 mAh g^−1^ was maintained after 60 cycles [122]. Similarly, LiI doping created additional Li^+^ transport channels, lowering activation barriers for S/Li_2_S conversion and delivering 85% capacity retention over 50 cycles at 0.2C [123]. Complementary halide‐doping approaches further enhance performance as small amounts of aliovalent/halide additives (AlI_3_ dissolved into Li_2_S) alter the local electronic/ionic structure and introduce defects/vacancies that raise Li^+^ mobility, lower overpotentials, and markedly improve capacity retention [122]. Adding LiI to conventional sulfide glass matrices (e.g., Li_2_S–P_2_S_5_) likewise increases room‐temperature Li^+^ conductivity (5.6 × 10^−4^ S·cm^−1^) and, on controlled crystallization, can promote high‐conductivity Li_7_P_3_S_11_‐type phases; however, LiI content must be optimized because excess halide can modify phase evolution and the electrochemical stability window [124]. In addition to conductive enhancement, electrolytes with redox‐mediating capabilities, such as LBPSI, have redefined interfacial chemistry. During charging, interfacial I^−^ is oxidized to I_2_/I_3_^−^, which chemically oxidizes Li_2_S at the Li_2_S/electrolyte boundary. This mediator‐enabled process activates previously inert interfaces, allowing capacities up to 1497 mAh g^−1^ at 2C and 80.2% retention after 25 000 cycles at 5C [98]. Improving the anode–electrolyte compatibility is also vital. To suppress lithium dendrite formation and enhance safety, lithium‐free anodes, such as silicon and graphite, have been explored. A core–shell Si@LPS anode, featuring a Li_7_P_3_S_11_ coating, effectively accommodated the approximately 400% volume expansion of silicon while promoting Li^+^ diffusion. The resulting full cell achieved 86.2% capacity retention after 35 cycles, significantly outperforming uncoated silicon (10.8%), with a theoretical energy density of 1495 Wh kg^−1^, which surpasses current lithium‐ion benchmarks [44]. Surface engineering of current‐collectors also plays a decisive role. Incorporating lithiophilic silver nanoparticles onto copper substrates promoted uniform lithium deposition, achieving a dendrite‐free morphology and extending anode‐free cell lifetimes to 200 cycles with a 98% Coulombic efficiency [102]. Additionally, a stainless‐steel mesh current‐collector combined with a Kevlar‐reinforced solid electrolyte (100 µm‐thick) enhanced mechanical strength and reduced interfacial resistance, delivering a cell‐level energy density of 370.6 Wh kg^−1^ at a 7.64 mg cm^−2^ Li_2_S loading [99]. Figure 8 summarizes these recent achievements, visually connecting structural design to electrochemical performance. Figure 8a schematically illustrates the fabrication of a supported Li_2_S cathode with a thin sulfide SSE, illustrating the layered integration of Li_2_S, conductive additives, and Kevlar fiber reinforcements for interfacial robustness [99]. Figure 8b shows a high‐resolution transmission electron microscopy (HRTEM) image of the Si@LPS anode, confirming that the uniform Li_7_P_3_S_11_ coating maintains intimate contact and buffers the mechanical expansion of silicon [44]. Figure 8c compares the interfacial stability through phenolphthalein indicator tests: although bare Li_2_S rapidly induced alkalization, the surface‐engineered Li_2_S@PD and Li_2_S@GPD samples maintained a neutral pH, validating the effectiveness of polymer‐gel coatings in suppressing side reactions [102]. Figure 8d shows the XRD profiles of Li_6_PS_5_Br electrolytes synthesized using the liquid‐phase and ball‐milling methods, demonstrating that liquid synthesis yields higher crystallinity and phase uniformity, correlating with superior ionic transport [124]. Figure 8e shows cycling performance of the (100 – *x*)Li_2_S·*x*Y_2_S_3_ active cathode materials (*x*  = 0, 0.5, 1, 2, 3, 4, and 5) at 0.05 C [101]. Figure 8f shows the galvanostatic charge–discharge curves of an ASSLSB employing a Li_2_S@C nanocomposite cathode, which shows stable voltage plateaus and high reversibility at 60°C under a practical areal loading [49]. Electrolyte engineering continues to be pivotal in all‐solid‐state architectures. The liquid‐phase synthesis of sulfide electrolytes offers superior particle homogeneity and a smaller grain size than ball milling. For example, Li_6_PS_5_Br synthesized in THF–EtOH yielded submicron particles (< 1 µm) with 10^−3^ S cm^−1^ conductivity. When integrated with Li_2_S‐LiI and vapor‐grown carbon fibers, this composite formed a 3D ion‐electron conductive network, achieving 1009 mAh g^−1^ at 0.05C and 650 mAh g^−1^ at 0.1C after 100 cycles [125]. Similarly, Li_7_P_3_S_11_ prepared via liquid‐phase routes improved electrode/electrolyte contact and reduced charge‐transfer resistance by approximately 40% relative to ball‐milled samples [126]. Hybrid designs incorporating polymers further balance the mechanical flexibility and ionic conductivity. A GO–interlinked poly(DOL) (GPD) gel electrolyte exhibited 1.1 mS cm^−1^ conductivity and a Li^+^ transference number of 0.68, effectively suppressing dendrite formation and polysulfide crossover. The resulting anode‐free quasi‐solid‐state cell achieved a volumetric energy density of 1093 Wh L^−1^, outperforming state‐of‐the‐art lithium‐ion systems [102]. Recent advances have emphasized 3D electrode architectures and in situ interface formation to maximize active mass utilization. A Li_2_S‐LiI‐mesoporous carbon composite (pore size ≈10 nm) integrated with vapor‐grown carbon fibers delivered 90% capacity retention after 100 cycles at a 5.78 mg cm^−2^ loading [125]. Similarly, microwave‐synthesized Li_2_S within 3D carbon scaffolds achieved a 440 mAh g^−1^ capacity retention over 400 cycles at 100 µA cm^−2^ [119]. Furthermore, in situ‐synthesized Li_3_PS_4_ coatings on Li_2_S‐acetylene black composites via ball milling formed conformal ion‐conducting layers, reducing interfacial resistance and increasing Li_2_S utilization to 90.9%, corresponding to an areal capacity of 5.52 mAh cm^−2^ [113].

## 4 Conclusions and Perspectives

Li_2_S has evolved from a difficult discharge product into a versatile design platform for next‐generation Li─S batteries, enabling safe and lithium‐ and anode‐free configurations. Future progress will depend on the concerted optimization of three inseparable levers, namely, interfacial catalysis, electrolyte and solvation design, and mesoscale electrode architecture, which collectively determine the activation barrier, reversibility, and manufacturability of Li_2_S. These advances must be realized under realistic system constraints, including lean electrolyte conditions (E/S ≤ 2.0), high sulfur loadings (4–7 mg cm^−2^), and low N/P ratios (N/P ≈ 1.0–1.2), all evaluated using standardized, device‐level metrics. At high mass loadings, the first‐charge activation of Li_2_S remains fundamentally kinetic and spatially heterogeneous, with polarization gradients exacerbated by limited ionic and electronic percolation in thick electrode. Catalytic motifs based on transition‐metal carbides, phosphides, sulfides, oxides, nitrides, or single‐atom M‐N_x_/S_x_ sites can reduce Li^+^ extraction barriers and stabilize transient polysulfide intermediates, while chemically bonded Li_2_S‐host interfaces suppress interfacial impedance growth. When combined with bi‐continuous, low‐tortuosity ion‐electron networks, these strategies can limit activation overpotentials to approximately 0.25–0.30 V at practical current densities, even at areal loadings ≥ 5 mg cm^−2^. Persuasive demonstrations will therefore require coupling operando kinetic descriptors such as Tafel slopes, apparent activation energies, and nucleation overpotential with durable cycling of thick electrodes over hundreds of cycles. Halide incorporation is also effective across materials classes. In particular, I^−^ substitution expands the Li_2_S lattice and increases Li–lattice distances and polarizability, lowering activation barriers for Li^+^ migration and directly enhancing macroscopic ionic conductivity in Li_2_S‐LiI solid solutions and doped systems. Beyond bulk chemistry, electrolytes should actively program the Li_2_S interface rather than merely conduct ions. Localized high‐concentration formulations with weakly solvating and fluorine‐rich co‐solvents, as well as redox mediators, can promote Li_2_S activation at approximately 2.2–2.5 V, suppress polysulfide shuttle, and form LiF‐rich mechanically robust interphases. Such electrolyte engineering is also central to enabling graphite‐Li_2_S full cells with a single electrolyte system. Conventional carbonate electrolytes, while compatible with graphite, are intrinsically incompatible with sulfur and polysulfide chemistry, whereas dilute ether electrolytes induce solvent co‐intercalation and graphite exfoliation. Localized high‐concentration or fluorinated ether‐based electrolytes offer a viable compromise, behaving effectively “carbonate‐like” at the graphite interface while preserving favourable Li_2_S redox kinetics. Under lean electrolyte and wide‐temperature operation (−10 to +45°C), success will be defined by activation voltages below 2.5 V, average Coulombic efficiencies of 99.7–99.9%, minimal swelling, and negligible self‐discharge. At the electrode level, architectures must accommodate the ∼80% S ↔ Li_2_S volume change while preserving electronic and ionic continuity. Hierarchical carbons, polar scaffolds, and elastic binders can deliver areal capacities > 5 mAh cm−^2^ with < 10% swelling and remain compatible with roll‐to‐roll manufacturing. Li_2_S/graphite full cells will be most manufacturable if the activation plateau remains below 2.5 V, capacity retention exceeds 80% after 500 cycles, and N/P stays within 1.2. Li_2_S/Si or SiO_x_ cells can achieve specific energies beyond graphite formats when swelling remains under 8% and areal capacities reach at least 4 mAh cm−^2^, while anode‐free designs become practical once plating efficiencies approach 99.9% with nucleation overpotentials below 20 mV. For all‐solid‐state architectures, composites capable of delivering ≥ 4 mAh cm^−^^2^ with interfacial resistance growth below 0.05 Ω·cm^2^ per cycle and < 20% capacity loss after 500 cycles will define meaningful progress. Scalable manufacture will depend on air‐stable Li_2_S composites protected by polymer or inorganic sheaths that resist ambient degradation, tolerate 8–24 h exposure, and form processable slurries with ≥ 60 wt.% solids for defect‐free coatings at pilot‐line speeds. Data comparability remains a critical bottleneck; reports should include areal loading, electrolyte‐to‐sulfur ratio, N/P ratio, activation method, electrolyte composition, voltage profiles, average Coulombic efficiency, gas evolution, and dimensional changes. Machine‐learning approaches linking DFT‐derived descriptors—such as S–p/d state coupling, adsorption energies, and ion diffusion barriers—to operando observables may eventually accelerate the discovery of device‐level metrics, including activation voltage, efficiency under lean electrolytes, and electrode swelling, although practical demonstrations remain limited. Quantitatively, the next three to five years should target activation overpotentials near 0.25 V, steady‐state charge overpotentials below 100 mV, minimum areal capacities of 5 mAh cm^−^^2^ at E/S ≤ 2.0 and N/P ≤ 1.2, and capacity retention > 80% after 500 cycles with average Coulombic efficiencies of approximately 99.9%. Graphite‐based full cells achieving 400–500 Wh kg^−^^1^ for over 300 cycles without co‐intercalation, anode‐free pouches exceeding 350 Wh kg^−^^1^ and 700 Wh L^−^^1^ initially with 200‐cycle retention to 80%, and solid‐state Li_2_S composites delivering ≥ 4 mAh cm^−^^2^ with < 20% loss after 500 cycles represent practical benchmarks. With catalysis‐guided activation, mediator‐assisted electrolytes, and strain‐tolerant architectures co‐optimized under manufacturing and sustainability constraints, Li_2_S can transition from an alternative cathode to a practical platform, redefining Li─S batteries as safe, high‐energy, and manufacturable systems for the post–lithium‐metal era.

## Conflicts of Interest

The authors declare no conflict of interest.

## Data Availability

The data that support the findings of this study are available in the supplementary material of this article.
